# Supplementary material for: Matrix Nanopatterning Regulates Mesenchymal Differentiation through Focal Adhesion Size and Distribution According to Cell Fate
Source: Biomimetics (Basel). 2019 Jun 25;4(2):43. doi: 10.3390/biomimetics4020043 (PMC6630613; doi:10.3390/biomimetics4020043)
Supplement: Supplementary file 1 [file biomimetics-04-00043-s001.pdf]

biomimetics

Supplementary information

Matrix Nanopatterning Regulates Mesenchymal Differentiation through Focal Adhesion Size and Distribution According to Cell Fate

Ignasi Casanellas [1,2], Anna Lagunas [3,1,*], Yolanda Vida [4,5], Ezequiel Pérez-Inestrosa [4,5], José A. Andrades [6,3], José Becerra [6,3,5] and Josep Samitier [1,2,3]

1. Institute for Bioengineering of Catalonia (IBEC), Barcelona Institute of Science and Technology (BIST), 08028 Barcelona, Spain.
2. Department of Electronics and Biomedical Engineering, University of Barcelona (UB), 08028 Barcelona, Spain.
3. Networking Biomedical Research Center in Bioengineering, Biomaterials and Nanomedicine (CIBER-BBN), 28029 Madrid, Spain.
4. Departamento de Química Orgánica, Facultad de Ciencias, Universidad de Málaga-IBIMA, 29071 Málaga, Spain.
5. Centro Andaluz de Nanomedicina y Biotecnología-BIONAND, 29590 Campanillas (Málaga), Spain.
6. Department of Cell Biology, Genetics and Physiology, Universidad de Málaga-IBIMA, 29071 Málaga, Spain.

* Correspondence: alagunas@ibecbarcelona.eu

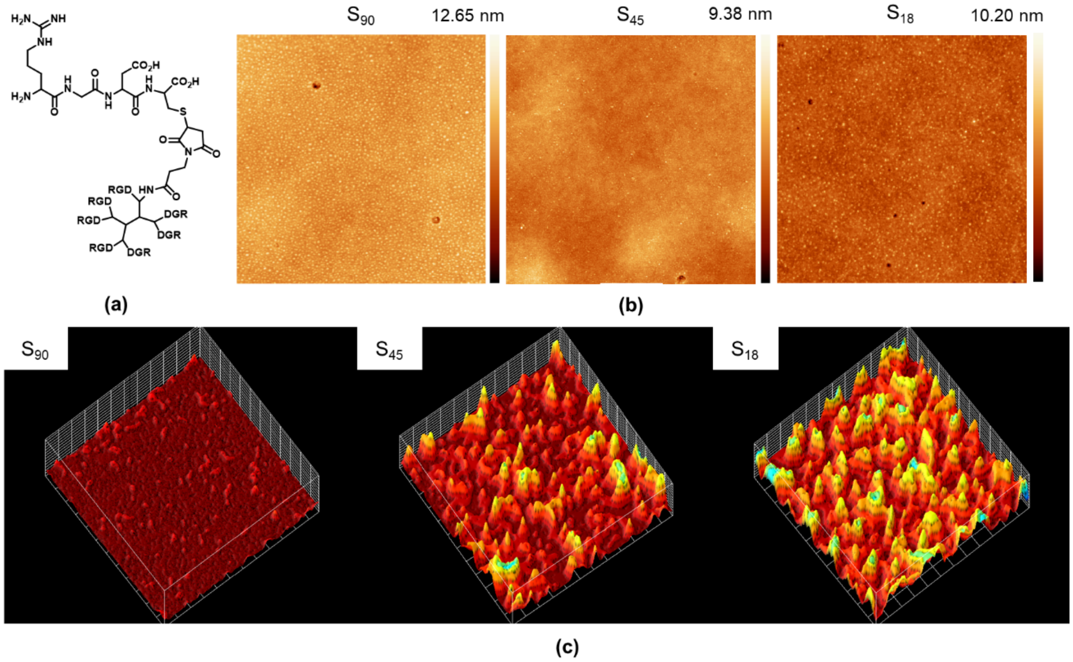

Supplementary Figure 1. Dendrimer-based RGD uneven nanopatterns that allow control on local surface adhesiveness at the nanoscale. (**a**) PAMAM G1-derived dendrimers containing eight copies of the cell-adhesive peptide RGD. (**b**) Representative AFM height images (5 × 5 μm) of the nanopatterns on PLLA obtained from initial aqueous solutions of 2.5 10^{-8}% (S_{90}), 1 10^{-8}% (S_{45}) and 4 10^{-9}% *w*/*w* (S_{18}), respectively and (**c**) the corresponding three-dimensional plots of minimum interparticle

distance (d_{min}) probability contour maps, showing high-density RGD regions (d_{min} < 70 nm) in dark red.

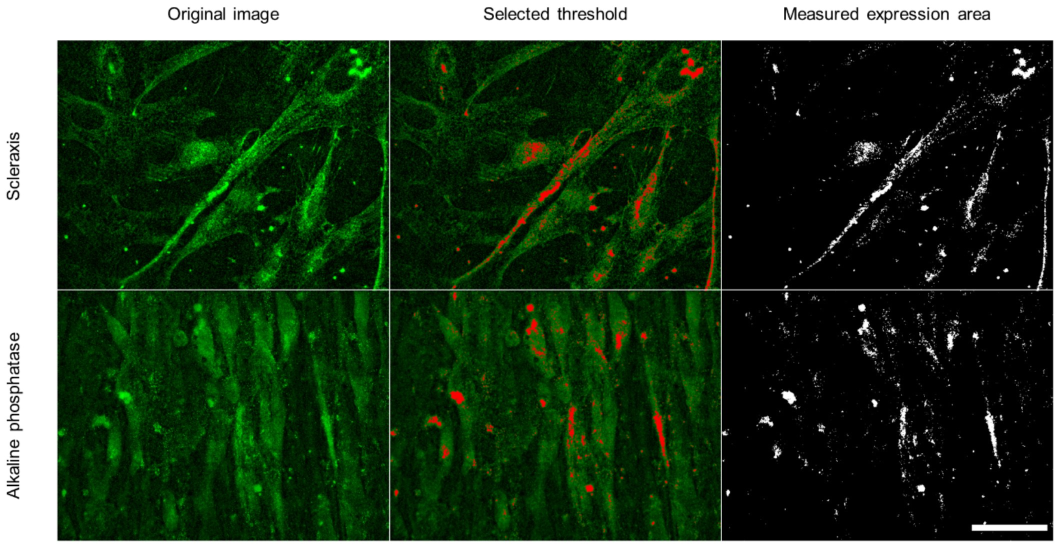

Supplementary Figure 2. Analysis of differentiation markers expression for tenogenesis (top) and osteogenesis (bottom). Immunostained images were treated, and the corresponding area of marker expression was selected and measured. Scale bar = 50 μm.

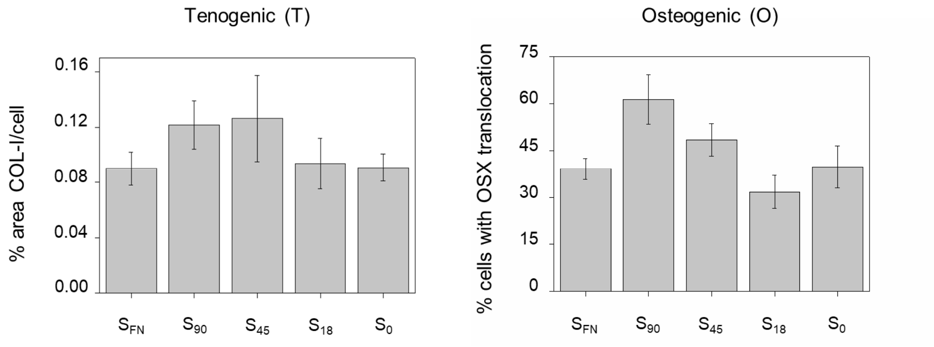

Supplementary Figure 3. Quantification of the percentage of area per cell on the different substrates of type-I collagen (COL-I) marker after three days of tenogenic (T) induction, and of the percentage of cells with osterix (OSX) translocation to the nuclei after 48 h of osteogenic (O) induction.

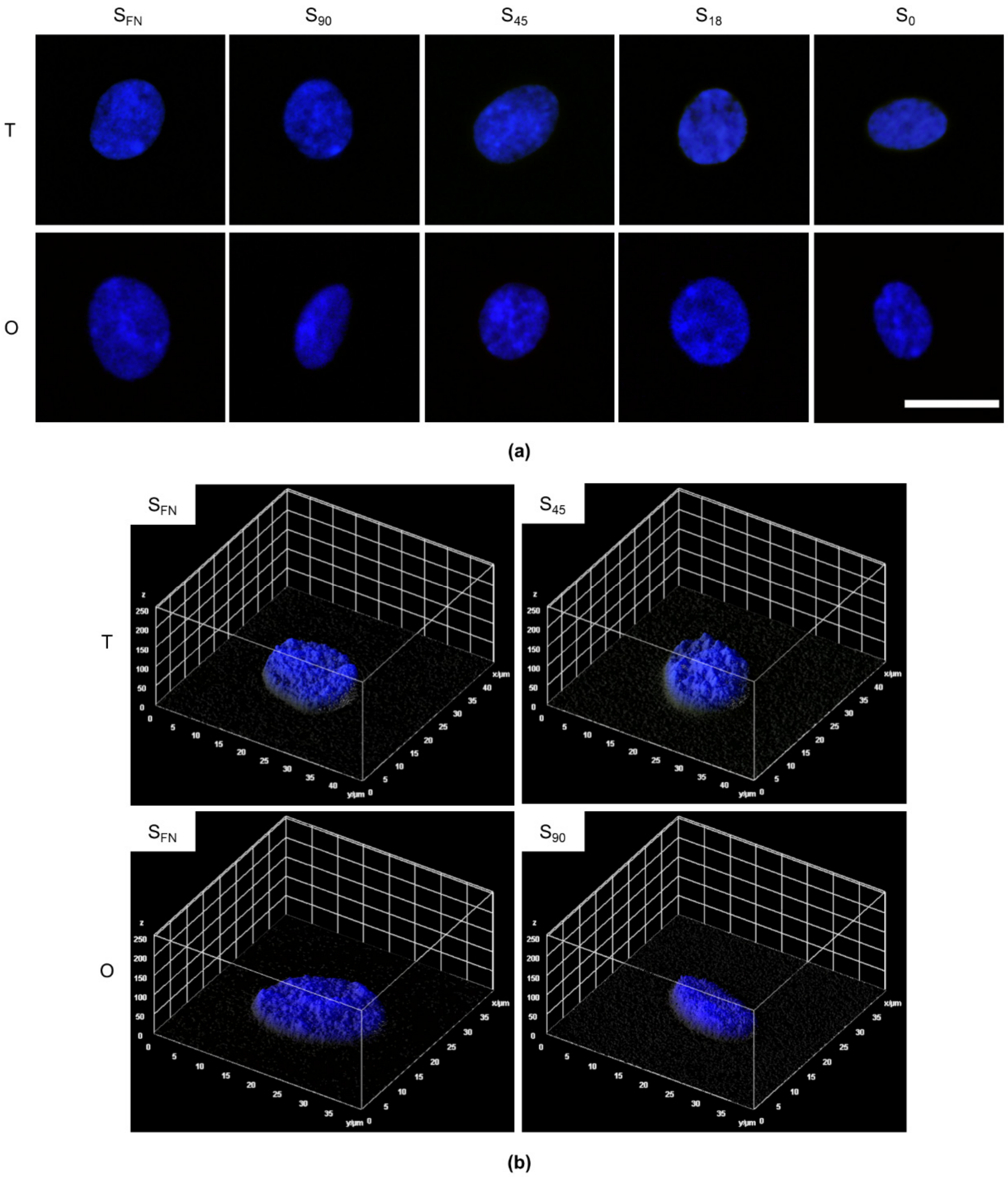

Supplementary Figure 4. Examination of chromatin condensation degree. (**a**) Representative epifluorescence images of cell nuclei (stained with Hoechst) obtained for hAMSCs cultured under tenogenic (T) or osteogenic (O) induction for 24 h. Scale bar = 20 µm. (**b**) Comparison of three-dimensional surface plots showing chromatin condensations (Hoechst) obtained on nanopatterns with highest nuclear elongation versus their respective positive controls under tenogenic and osteogenic stimulation. No significant differences were found.
